# Myxoma of the Ovary: in Regard to Bedir et al. (Balkan Med J 2018;35:120-121)

**DOI:** 10.4274/balkanmedj.2018.1454

**Published:** 2019-01-01

**Authors:** Nicolas Sterkers, Francis Salvat, Philippe Sappa, Olivier Donnez

**Affiliations:** 1 Department of Cancerology and Gynecology Surgery, Urbain V Clinic, Avignon, France; 2 Radiology Center, Scanner Clinique Fontvert Urbain V, Sorgues, France; 3 Pathology Center, Histosud, Chemin De Faufinette, Valreas, France

To the Editor,

In the January 2018 issue of Balkan Medical Journal, Bedir et al. (1) reported a new case of an ovarian myxoma, an exceedingly rare ovarian tumor. Although ovarian myxoma is a benign tumor that occurs predominantly in young women in whom fertility preservation is a major concern, the authors suggest that total excision of the tumor together with the adnexal structures is advisable because of the difficulty of total excision of viscous material that may cause recurrences. Here we describe a case wherein safe and efficient ovarian-sparing surgery was offered to a young woman with an ovarian myxoma, and we suggest this approach as a valuable option to treat this benign tumor.

A 22-year-old woman, 0 gravida, with an abdominal mass was referred to our department. Her medical history was characterized by achondroplasia with hydrocephalus that was cured by ventriculoperitoneal shunting in early childhood. Gynecological history was uneventful. Ultrasound and subsequent computed tomography of the abdomen after contrast administration revealed a well-defined cystic lesion (8/10.5/11 cm) with a thin capsular wall in the right iliac fossa. The tumor was adjacent to the ventriculoperitoneal-shunt catheter but inextricably linked to the right ovary, which argued against the diagnosis of a pseudocyst related to ventriculoperitoneal shunting complication. The CA-125 biomarker showed a normal value (28.5 IU/mL). A multidisciplinary consultation predicted the tumor to be benign, and a laparoscopic fertility-sparing surgery was scheduled. An exploratory laparoscopy revealed that the tumor was a well-circumscribed cystic mass arising from the right ovary, free of adjacent structures, with an external gray, translucent, and smooth surface. Given the normally looking abdomen, the absence of abnormal nodes, and the need for optimal fertility preservation, complete cystectomy preserving both ovaries was selected as the therapeutic approach. The cyst section revealed cystic spaces filled by gelatinous material. After aspiration of the cyst, the incision was enlarged. Blunt and sharp dissection helped to find a cleavage plane between the cyst wall and the ovarian cortex. The entire tumor was removed using an endobag to avoid tumor spillage. The peritoneal cavity was washed with saline, and the fluid was aspirated at the end of the procedure. Pathological examination revealed abundant myxoid stroma with positive staining with alcian blue, limited fibrotic spaces, and interspaced spindle-shaped tumor cells without atypia, of which approximately half stained with alpha-smooth muscle actin. Staining for S100 protein, keratin (AE 1/3), and inhibin was negative. Ki67 expression was low (3-4%). The diagnosis of ovarian myxoma was made and confirmed by the French National Cancer Institute committee. No recurrence was detected even 6 years after the operation. The patient provided a written informed consent for publication.

Data on ovarian myxoma management are scarce and mostly extrapolated from isolated case reports or small histopathological series. This rare tumor was first mentioned in 1960 in a 14-year-old case. In 1991, Eichorn reported five personally observed cases and reviewed three additional previously described cases (2). We found seven additional isolated cases with acceptable, although limited, clinical documentation. Thus, a total of 15 cases have been reviewed till date. Ovarian myxoma frequently occurs in women at the reproductive age (9/16 cases, including ours, were aged ≤40 years), with a peak of incidence lying between ages 12 and 25 years (n=9/16, 56%). The available clinical characteristics, management, and the outcome of the nine cases aged ≤40 years (1,2,3,4,5) are summarized in the Table 1. Ovarian masses in young women require a thoughtful consideration to the preservation of the ovary if the preoperative suspicion of malignancy is low and there is no evidence of malignancy intraoperatively (6,7,8,9,10). Nevertheless, as shown in the table, ovarian myxoma has traditionally been removed through unilateral oophorectomy even in women at the reproductive age (n=8/8). Furthermore, laparoscopy that has been established as an adequate alternative to laparotomy for the treatment of benign ovarian tumors (11) reduces postoperative adhesion formation that may compromise fertility. Safe and efficient treatment of our case by laparoscopic ovarian-sparing surgery suggests that this approach is convenient for adolescent and young women in whom any effort should be made to preserve fertility. However, two primary considerations should still be taken into account. The tumor must be presumed to be at low risk based on careful preoperative and operative evaluation. When this rare tumor is postoperatively diagnosed by histopathological examination, the surgeon must be adequately trained in oncological procedures to avoid tumor spillage (12).

## Figures and Tables

**Table 1:**
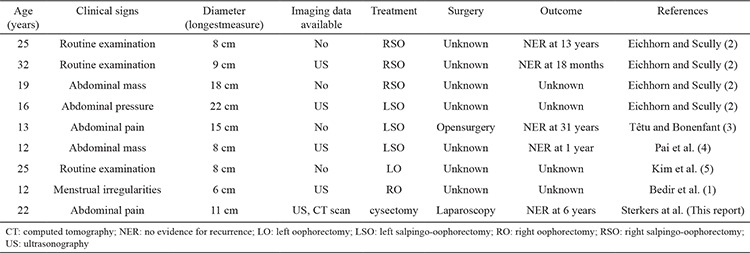
Primary clinical characteristics of eight cases aged ≤40 years reported in the literature and our case
